# Comparison of fieldworker interview and a pictorial diary method for recording morbidity of infants in semi-urban slums

**DOI:** 10.1186/s12889-015-1372-7

**Published:** 2015-01-31

**Authors:** Rahul Jacob Thomas, Karthikeyan Ramanujam, Vasanthakumar Velusamy, Saravanakumar Puthupalayam Kaliappan, Deepthi Kattula, Jayaprakash Muliyil, Gagandeep Kang

**Affiliations:** Division of Gastrointestinal Sciences, Christian Medical College, Vellore, TN 632004 India

**Keywords:** Reliability, Children, Morbidity measurements, Slum, India, Fieldworker, Pictorial diary, Kappa, Agreement

## Abstract

**Background:**

Cohort studies conducted in low-income countries generally use trained fieldworkers for collecting data on home visits. In industrialised countries, researchers use less resource intensive methods, such as self-administered structured questionnaires or symptom diaries. This study compared and assessed the reliability of the data on diarrhoea, fever and cough/cold in children as obtained by a pictorial diary maintained by the mother and collected separately by a fieldworker.

**Methods:**

A sample of 205 children was randomly selected from an ongoing birth cohort study. Pictorial diaries were distributed weekly to mothers of study children who were asked to maintain a record of morbidity for four weeks. We compared the reliability and completeness of the data on diarrhoea, fever and cough/cold obtained by the two methods.

**Results:**

Of 205 participants, 186 (91%) ever made a record in the diary and 62 (30%) mothers maintained the diary for all 28 days. The prevalence-adjusted bias-adjusted kappa statistics for diarrhoea, fever, cough/cold and for a healthy child were 92%, 79%, 35% and 35% respectively.

**Conclusion:**

Diary recording was incomplete in the majority of households. When recorded, the morbidity data by the pictorial diary method for acute illnesses were reliable. Strategies are needed to address behavioural factors affecting maternal recording such that field studies can obtain accurate morbidity measurements with limited resources.

## Background

Quality of data is critically important in any study. Several aspects of epidemiological data collection such as completeness, clarity, interviewer’s skill and education level of the responder determine the quality of data [1]. Cohort studies measure exposure factors at different time points [2] to evaluate the association between exposure and disease. Cohort studies in low-income countries largely use trained fieldworkers to make frequent visits to a participant’s home for routine surveillance, as opposed to industrialised countries where less resource intensive approaches, such as mailing self-administered structured questionnaires or maintaining a diary to record day to day morbidity are possible. Diarrhoea surveillance programs often employ both interviews and diary methods to record morbidity and obtain data on severity of illness [3].

The fieldworker interview has many advantages like reliability, validity and high response rate but incurs costs in terms of training, travelling and time which can have considerable impact on study size and the need for resources [4]. Diary methods and self-administered questionnaires are more effective and beneficial in populations with high literacy levels and are not always recommended in low literacy level settings [5,6], but where used, are suggested for daily events [5,7-9].

We hypothesised that morbidity data collected through a pictorial diary method maintained by the mother would be as good as fieldworker records on a structured questionnaire used at home visits. The study compared the morbidity data recorded by a pictorial diary method against the data collected by the fieldworkers and assessed reliability of data collection for diarrhoea, fever and cough/cold.

## Methods

### Study setting, participants and data collection

**Figure 1:**
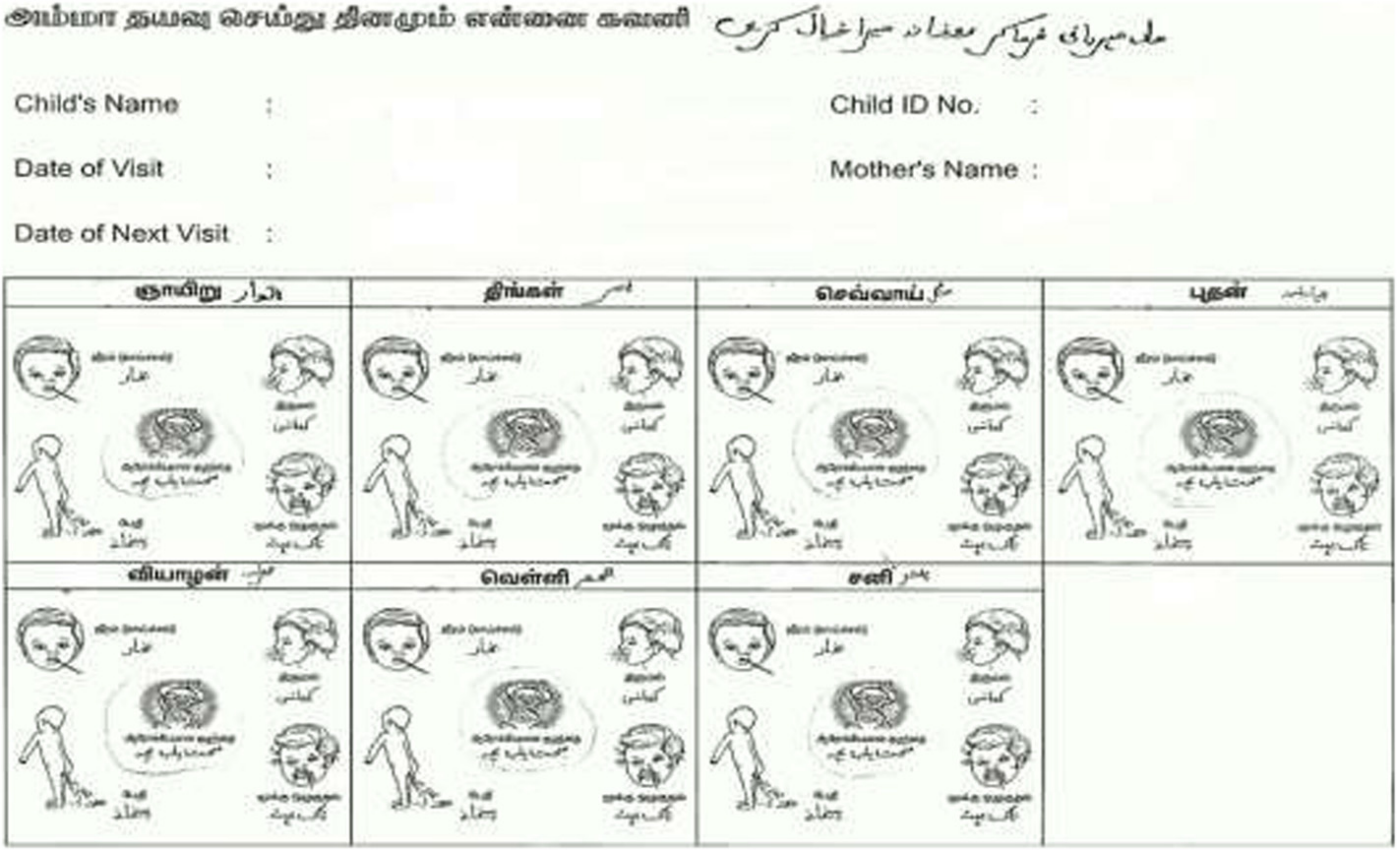
**Pictorial diary for seven days showing (clock wise) a child with symptoms of fever, cough, cold and diarrhoea and a healthy child in the centre.** Mothers were asked to circle the appropriate picture each day. Descriptions are written in Tamil and Urdu, the local languages.

The study was conducted from April 2010 to May 2010. The participants were the mothers of children enrolled in an ongoing cohort that studied the natural history and immune response to *Cryptosporidium* spp. in children from birth to 3 years of age in the semi-urban slums of Vellore. The study was approved by the Institutional Review Board of the Christian Medical College, Vellore, India and written informed consent was obtained from the parents. A description of the study setting [10] and of the morbidity definitions (Table 1) have been published [11]. For this study we used data on diarrhoea, fever, and cough/cold illness which were depicted in the easy to use pictorial diary (Figure 1).

**Table 1:** **Definitions used by the fieldworkers and mothers in assessing illness among study children**

**Symptom/illness**	**Definition**	**Duration**	**Time interval for a new episode**
Diarrhoea	Three or more watery stools per day or a change in number or consistency reported by the mother and which she considers indicative of diarrhoea	≥1 day	48 hrs after cessation of the previous episode
Fever	Increased temperature of the body as perceived by primary caregiver	≥2 days	72 hrs after cessation of the previous episode
Cough/cold	Cough/runny nose with or without fever	≥5 days	72 hrs after cessation of the previous episode

We estimated that approximately 200 subjects would be needed to provide 90% power to test the null hypothesis of kappa = 0.4 versus the alternate hypothesis kappa = 0.6 at 0.05 significance level (2 sided). A total of 205 subjects were selected by simple random sampling method using the cohort study database as a sampling frame and the pictorial diaries were distributed to their mothers by a separate set of fieldworkers not involved in the main cohort study. The purpose of the study and the definitions used (e.g. diarrhoea consists of 3 or more looser than normal stools in a 24 hour period) were explained to the mothers, with instructions to mark the child as healthy if there was no cough/cold, fever or diarrhoea. For 4 weeks, the fieldworkers visited the study mothers once a week, collected the previous week’s pictorial diary and handed over the next week’s pictorial diary.

The main cohort study fieldworkers were masked about the identity of pictorial diary participants and they continued their biweekly surveillance per the main study protocol. The morbidity data collected for the 205 children during the study period by the fieldworkers were extracted from the original study database for analysis. The fieldworker collected data were subjected to a 10% random recheck by a field supervisor through the duration of the study.

### Data entry

Double data entry was done and verified using Epi-Info 3.5.1 (CDC, GA, USA) for data collected in the diaries. The socio-demographic characteristics and surveillance data were extracted from the main study database. SPSS 16 (SPSS Inc., IL, USA) and STATA 10 (StataCorp, TX, USA) software were used for analysis.

### Statistical analysis

A test of association between baseline characteristics and completeness of the diary was performed using Pearson’s chi-squared test. McNemar’s chi-squared test was performed to compare correlated proportion of reported days of illness by both methods; p < 0.05 was considered to be statistically significant. The reliability of number of child illness days was calculated using kappa statistics and prevalence-adjusted bias-adjusted kappa (PABAK) [12]. In order to interpret kappa meaningfully it is important to report prevalence and bias along with kappa and provide adjusted kappa. A higher prevalence index results in lower kappa whereas higher bias index results in higher kappa and PABAK is used to adjust these paradoxes and the interpretation of the strength of agreement is the same as kappa [13,14]. For agreement on episodes of illness, percent agreement was used [15].

## Results

### Baseline characteristics

Of 205 mothers, 186 (91%) provided morbidity information in the diary for a period which ranged from 3 to 28 days (mean = 21 days, SD = 7 days). Nearly a third (62, 30%) of the mothers completed all the 28 days. Morbidity data was missing for 168 (3%) child-days in the fieldworker records due to non-availability of the primary caregiver on the day of scheduled follow up. For the 20 subjects where caregivers were not available to the field workers, complete diary data was available for 4 subjects (17 child-days), incomplete data was available for 5 subjects (16 of 56 child-days) and data was missing for 11 subjects (95 child-days). Overall, a total of 5572 child-days of observation in 205 children and 3897 child-days of observations in 186 children were available for morbidity assessment from the fieldworker and diary data, respectively. This information is presented in Table 2 and the selected baseline characteristics are presented in Table 3.

**Table 2:** **Child-days of follow up by the fieldworker and by the mothers with the pictorial diary**

	**Child-days (Number of subjects)**	
	**Fieldworkers**	**Mothers**
Number of child-days of follow up	5572 (205)	3897 (186)
Number of child-days completed for all 28 days	5180 (185)	1736 (62)
Comparison of missing child-days of follow up		
Number of child-days completely missed	168 (20)	95 (11)
Number of child-days recorded	-	17 (4)
Number of child-days partially recorded	-	16 (5)

**Table 3:** **Characteristics of the mothers and children who participated in the study**

**Variables**	**N = 205 n (%)**
**Age of the mother**	
More than 24 years	71 (35)
Less than or equal to 24 years	134 (65)
**Number of years of mother’s education**	
More than 5 years	92 (45)
Less than or equal to 5 years	113 (55)
**Socio economic status**	
Low	128 (62)
Middle and High	77 (37)
**Religion**	
Hindu	104 (51)
Muslim	92 (45)
Christian	9 (4)
**Family type**	
Joint	38 (19)
Extended	51 (25)
Nuclear	116 (57)
**Sex of the child**	
Male	104 (51)
Female	101 (49)
Birth weight of the child (N = 203*)	
Greater than or equal to 2500 grams	162 (80)
Less than 2500 grams	41 (20)
**Birth order of the child**	
Third or later born	55 (27)
First or second	150 (73)
**Age of the child**	
More than 6 months	97 (47)
Less than or equal to 6 months	108 (53)

*Birth weight of 2 subjects not available.

We did not find any significant difference among the mothers who completed (n = 62) and who did not complete (n = 124) the diary in age, education of the mother, socioeconomic status (SES) of the family, birth order of the child and family size.

### Illness reporting, inter-rater agreement and episodes

The number of reported days of illnesses by either method is shown in Table 4. The reporting by mothers was most complete for diarrhoea and least for cough/cold. The PABAK statistics were 92%, 79% and 35% for diarrhoea, fever and cough/cold respectively. The prevalence index, bias index [12] unadjusted kappa and PABAK are presented in Table 5.

**Table 4:** **Illness reported by fieldworker interview and by a pictorial diary maintained by the mother for 4 weeks in 186 children for whom data were available for any length of time by both methods**

**Illness**	**Number of days reported**	
	**Fieldworkers (%)**	**Mothers (%)**
Diarrhoea	128 (3)	112 (3)
Fever	389 (10)	232 (6)
Cough and cold	1917 (49)	993 (25)
>1 symptom reported	354 (9)	130 (3)
None	1843 (47)	2695 (69)

**Table 5:** **Reliability of two methods using Kappa statistics for observations reported by both methods (3897 observations in 186 children)**

**Variable**	**P**_0_	**P**_e_	**PI**	**BI**	**Kappa**	**BAK**	**PABAK**
Diarrhoea	0.962	0.940	−0.938	0.004	0.360	0.364	0.924
Fever	0.896	0.853	−0.841	0.040	0.290	0.291	0.792
Cough/cold	0.674	0.504	−0.253	0.237	0.342	0.302	0.347
Healthy	0.674	0.490	0.164	−0.219	0.361	0.329	0.347

P_o_ –Proportion observed.

P_e_-Proportion expected.

PI = Prevalence index (difference in the probability of agreement cells (a-d)/N).

BI = Bias index (difference in proportion of disagreement cells (b-c)/N).

BAK is Bias-adjusted kappa. It is calculated by substituting the average values of the discordant cells instead of their original values in the regular kappa estimation formula. K = (P_o_- P_e_)/ (1-P_e_).

PABAK = Prevalence-adjusted bias-adjusted kappa = 2*(P_o_)-1.

In the subset of subjects who had all the data for 28 days by both methods, the reported days of illness by fieldworkers were higher than reported by mothers: 61, 144 and 910 and 52, 108 and 420 days of illnesses for diarrhoea, fever and cough/cold respectively. These differences were evaluated using McNemar’s chi-squared test which was statistically significant for fever (p = 0.0036) and cough/cold (p < 0.001) but not for diarrhoea (p = 0.3425). Percent agreement of episodes of illnesses between fieldworker and diary data is presented in Table 6.

**Table 6:** **Percent agreement examining paired observations of episodes of illnesses between fieldworker and diary data**

	**Field worker**	**Calendar**			**Percent agreement**
ᅟ	ᅟ	**Reported**	**Not reported**	**Total**	
**Diarrheal episodes**	Reported	10	4	14	
	Not reported	6	-	6	50%
	Total	16	4	20	
**Fever episodes**	Reported	27	12	39	
	Not reported	13	-	13	52%
	Total	40	12	52	
**Cough/cold episodes**	Reported	27	27	54	
	Not reported	0	-	0	50%
	Total	27	27	54	

## Discussion

Biweekly morbidity surveillance data collected by fieldworkers were used to test the reliability of data reported daily in a pictorial calendar by the mothers. While 91% of the mothers had made at least one entry, only 30% of the mothers completed the diary for the entire 28 day period. No baseline difference could be identified for mothers who completed or did not complete the diaries.

A community based clinical trial in Australia which aimed at a high level of completion of a health diary over 68 weeks and a study in Canada to test the validity and feasibility of diary data collected by parents at certain times after vaccination and up to 21 days reported very high levels of completeness [16,17]. Diary methods are generally recommended for populations with adequate literacy levels [6,18], but a pictorial diary does not require literacy. Pictorial diary methods have been used in health utilisation, health expenditure and morbidity surveys [19-22]. Although maintaining a diary for long periods could result in fatigue and attrition [23], some studies suggest that encouraging the participants to improve compliance would result in improved reporting both in terms of accuracy and completion [7,9].

Among the reported illnesses, diarrhoea and fever showed considerable overlap while cough/cold had more reports by fieldworkers than in the diaries. One explanation could be under-reporting by mothers of common minor illnesses, as up to 7 respiratory illnesses per child per year have been previously reported in this setting [10].

Mothers reporting more diarrhoeal and fever episodes and fewer cough/colds (Table 6) could be attributed to sporadic marking in the diary for specific episodes, especially for longer duration episodes recorded by the field workers. This resulted in more than one episode being recorded instead of a single long episode if there were two or more missing days for diarrhoea and three or more missing days for fever. Since coughs/colds needed to be recorded for five or more days to count as an episode, the lack of marking in the diary resulted in fewer episodes than compared to fieldworkers’ records.

Reliability assessment (Table 5) shows that for diarrhoea and fever the proportion observed, chance agreement and prevalence index are high which resulted in lower kappa and when adjusted for the two paradoxes [24] bias and prevalence, the PABAK shows substantial agreement [14] for diarrhoea and fever respectively. An Argentine study reported poor kappa agreement of mother’s perception captured through a written questionnaire about their overweight and obese children compared to body mass index z-score [25] whereas a Bangladesh study showed that 60% of the mothers correctly identified malnutrition in their children [26]. A cross sectional study in Kenya reported that a pictorial method identified malnutrition better than verbal description though both methods under-estimated malnutrition when compared to formal anthropometric measurements [22]. The published literature on the use of pictorial methods in low-income settings therefore indicates that the pictorial diary method could be effective in recording illnesses if high rates of compliance could be achieved. Although only 30% of mothers recorded 4 weeks of data, their data were reliable and comparable to conventionally collected data, which suggests that even though diaries are vulnerable to attrition and fatigue they do not compromise the quality of data, as has been reported previously [7,8,23].

### Limitations

In order to avoid response bias which is one of the limitations of diary methods [27], the field workers did not insist that the mothers should complete the diaries and just collected the weekly pictorial diaries and issued blank ones. This study therefore assessed spontaneous diary completion, but reasons for not completing the diaries were not collected, which is the main limitation of this study.

## Conclusion

There was a high rate of attrition in pictorial diary use by mothers over a 4 week period. Where collected, the morbidity data recorded by pictorial diary methods for acute illnesses was reliable when compared to fieldworker collected data. Reasons why mothers did not complete diaries were not collected. Future studies should examine behavioural factors affecting motivation to complete diaries as a possible strategy to improve data collection in resource limited studies.
